# Characterization of an Enantioselective Odorant Receptor in the Yellow Fever Mosquito *Aedes aegypti*

**DOI:** 10.1371/journal.pone.0007032

**Published:** 2009-09-15

**Authors:** Jonathan D. Bohbot, Joseph C. Dickens

**Affiliations:** United States Department of Agriculture, Agricultural Research Service, Henry A. Wallace Beltsville Agricultural Research Center, Plant Sciences Institute, Invasive Insect Biocontrol and Behavior Laboratory, Beltsville, Maryland, United States of America; University of California Davis, United States of America

## Abstract

Enantiomers differ only in the left or right handedness (chirality) of their orientations and exhibit identical chemical and physical properties. In chemical communication systems, enantiomers can be differentially active at the physiological and behavioral levels. Only recently were enantioselective odorant receptors demonstrated in mammals while their existence in insects has remained hypothetical. Using the two-microelectrode voltage clamp of Xenopus oocytes, we show that the yellow fever mosquito, *Aedes aegypti*, odorant receptor 8 (AaOR8) acts as a chiral selective receptor for the (*R*)-(—)-enantiomer of 1-octen-3-ol, which in the presence of other kairomones is an attractant used by blood-sucking insects to locate their hosts. In addition to steric constraints, chain length and degree of unsaturation play important roles in this recognition process. This is the first characterization of an enantioselective odorant receptor in insects and the results demonstrate that an OR alone, without helper proteins, can account for chiral specificity exhibited by olfactory sensory neurons (OSNs).

## Introduction

Chiral specificity is certainly the most remarkable accomplishment of olfactory systems. From the elephant and beetle chiral pheromone frontalin [1] to the enantioselective abilities of squirrel monkeys [2], examples of chiral signals abound. Since the early 1970s, enantioselectivity of insect [3] olfactory systems has been well documented. Evidence ranges from enantiomer driven behaviors [4], [5], [6], [7], [8] and glomerular activation patterns [9] to highly specific olfactory receptor cells for pheromones and plant odorants [10], [11]. In fact, behavioral studies carried out in humans [2], honeybees [12] and mice [13] have clearly demonstrated the ability of these organisms to distinguish between chiral odorants, prompting several authors to postulate the existence of enantioselective ORs. Recently, one report has presented direct evidence that some mice ORs can discriminate odorant enantiomers [14]. As insect and vertebrate *Or* genes are phylogenetically unrelated [15], limited data exist at the molecular level for discrimination of enantiomers by insect ORs [16].

Insect OSNs typically express a combination of a member of the conventional OR family and a ubiquitously expressed and highly conserved co-receptor [17], [18], [19]. While the exact composition of this heteromeric complex remains unknown, it is apparent that the interaction between a variable odorant-binding OR and an obligatory partner protein called OR7 in mosquitoes [20], [21], [22], 83b in flies [17], and OR2 in bees [23] and moths [24], [25] is necessary to create a functional ion channel [26] and perhaps activate a G-protein pathway [27].

**Figure 1:**
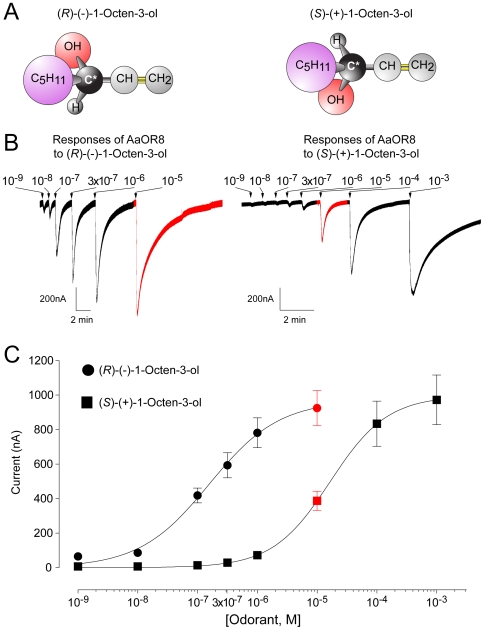
AaOR8 discriminates between the two enantiomers of 1-octen-3-ol. (A) The odorant 1-octen-3-ol occurs in two configurations: (*R*) and (*S*). Asterisk indicates the chiral center. (B) Response traces of AaOR8 to each enantiomer are recorded in nano-ampere (nA). For space considerations, time scales differ. (C) Concentration-response plots of AaOR8 to each enantiomer of 1-octen-3-ol (n = 6). Odorant concentrations were plotted on a logarithmic scale. Each point represents the mean and vertical current response; error bars are s.e.m. Responses to 10^−5^ M 1-octen-3-ol are highlighted in red.

Racemic 1-octen-3-ol (CH3[CH2]4CH[OH]CH = CH2) is a mono-unsaturated 8-carbon alcohol with carbon 3 being the single stereogenic center (Fig. 1A), hence its composition of two optically active enantiomers, (*R*)-(—)-1-octen-3-ol and (*S*)-(+)-1-octen-3-ol. Octenol is a natural compound of plant [28] and animal origin [29], and has been identified from human sweat extracts [30]. (*R*)-(—)-1-octen-3-ol is the prevailing enantiomer in volatiles collected from cattle with a (*R*)/(*S*) ratio between 80% and 92% [29]. While both octenol enantiomers are equally active aggregation pheromones for several beetle species [31] and potent attractants to the tsetse fly, *Glossina morsitans* [29], many mosquito species exhibit a preference for the (*R*)-(—) form [32], [33]. This compound alone is an attractant for various hematophagous insects [29], [34] and its behavioral potency is increased when combined with CO_2_ [35]. OSNs located within the capitate peg sensilla on the maxillary palps of *Aedes aegypti* [36], *Culex quinquefasciatus* [33] and *Anopheles gambiae* [16] mediate the response to octenol and CO_2_. In the case of *An. gambiae*, the molecular basis of the octenol response has previously been attributed to *An. gambiae* OR8 (AgOR8) [16]. We recently identified the *Or* gene family of *Ae. aegypti* including the *Ae*. *aegypti* orthologue of *AgOr8*, *AaOr8* [37]. In the current study, we establish that octenol is the preferred ligand of the AaOR8/AaOR7 protein complex, and investigate the structure-activity relationship between ligand and receptor, focusing on the enantiomeric discrimination of (*R*)- and (*S*)-octenol.

## Results

### AaOR8 is activated by (*R*)-(—)-1-octen-3-ol

Along with *AaOr8*, we co-expressed *AaOr7* in Xenopus oocytes and electrophysiological responses (Fig. 1B) to each enantiomer were measured using the two-microelectrode voltage clamp technique. In contrast to AaOR8, which belongs to the highly divergent class of ORs, members of the insect OR7 family exhibit high sequence homology and associate with conventional ORs to form a functional hetero-complex [15]. We established the concentration-response relationships for each compound (Fig. 1C) and their associated half maximal effective concentration (EC_50_) as a sensitivity criterion. AaOR8 was most sensitive to the (*R*)-(—)-1-octen-3-ol with an EC_50_ value of 158 nM, two orders of magnitude lower than to the (*S*)-(+)-enantiomer (EC_50_ = 17,200 nM). Part of the response to the (*S*)-(+)-enantiomer may have been caused by the presence of trace amounts (1 part per thousand) of the (*R*)-(—)-enantiomer (see Material & Methods).

### Effect of the chiral center on AaOR8 activation

**Figure 2:**
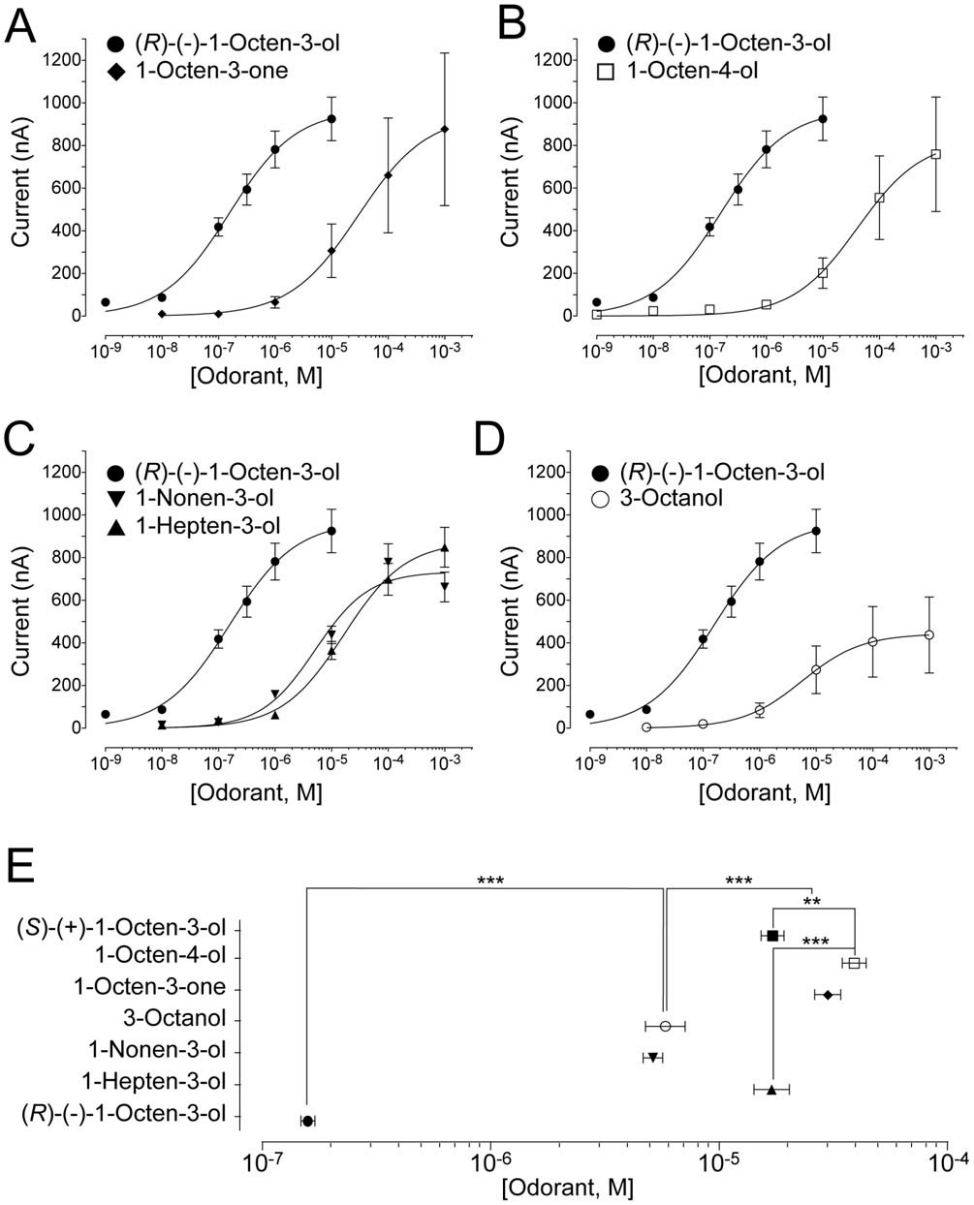
Strong preference of AaOR8 towards (*R*)-(—)-1-octen-3-ol. The concentration-response plot for (*R*)-(—)-1-octen-3-ol was repeated in each panel for comparative purposes. (A) Importance of C3 as a chiral center. Concentration-response plots of AaOR8 to 1-octen-3-one (n = 6). (B) Shifting the chiral center from C^3^ to C^4^ reduces AaOR8 sensitivity. Concentration-response plots of AaOR8 to 1-octen-4-ol (n = 8). (C) Side chain length affects AaOR8 sensitivity. Concentration-response plots of AaOR8 to 1-nonen-3-ol and 1-hepten-3-ol (n = 8 to 9). (D) The double bond is critical for recognition by AaOR8. Concentration-response plots of AaOR8 to 3-octanol (n = 6). (E) EC_50_ ranking profile of AaOR8 for octenol related compounds. Asterisk, p<0.05; two asterisks, p<0.01 and three asterisks, p<0.001. Odorant concentrations were plotted on a logarithmic scale. Each point represents the mean and error bars indicate s.e.m.

**Figure 3:**
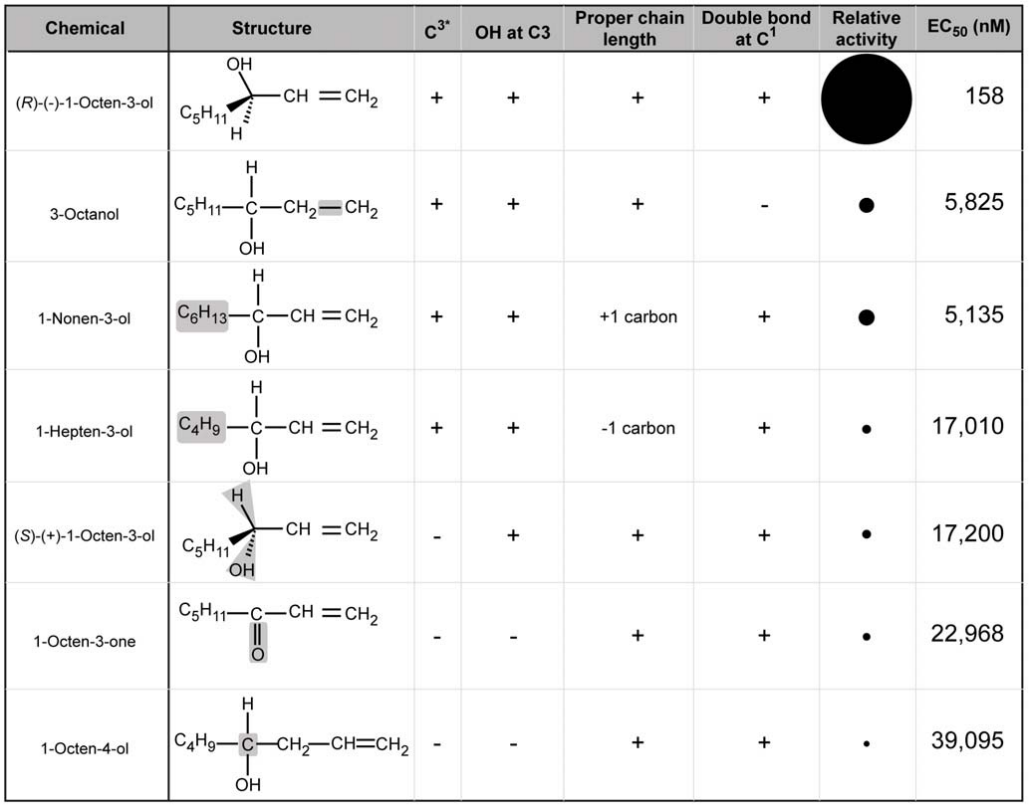
Relative activity of AaOR8 towards (*R*)-(—)-1-octen-3-ol and related compounds. Changes relative to (*R*)-(—)-1-octen-3-ol are shaded in grey. Presence and absence of a specific chemical feature are indicated by + and -, respectively. The formula of racemic compounds does not assume the 3 dimensional orientation of the residues attached to the chiral center. Note that the least active isomers are those lacking the proper chirality at the 3-position (C^3*^). Relative activities of each odorant (EC_50s_) are reflected by area of solid circles.

Deduced EC_50_ ranking agonist profiles were used to further evaluate the importance of the chiral center in this recognition process (Figs. 2 and 3). Replacing the hydroxy moiety of octenol by a ketone group, rendering the molecule achiral, reduced AaOR8 sensitivity by over two log steps (Fig. 2A and E). Displacing the chiral center to position C^4^ had a similar effect on AaOR8 sensitivity (Fig. 2B and E).

### Chain length and unsaturation are required for AaOR8 activation

Chain length and degree of unsaturation also proved to be important determinants for AaOR8 sensitivity (Fig. 2, C and D). Adding or removing one carbon lowered the response of AaOR8, although in an asymmetric fashion. AaOR8 displayed higher sensitivity to the longer C9 alcohol, 1-nonen-3-ol, than to the shorter C7 alcohol, 1-hepten-3-ol (Fig. 2, C and E). Lower sensitivity to 1-hepten-3-ol had already been shown in the case of AgOR8 [16]. Unsaturation on carbon 1 was of consequence as the saturated 3-octanol compound decreased AaOR8 sensitivity 10-fold compared to (*R*)-(—)-1-octen-3-ol (Fig. 2D and E), a result consistent with previously reported electrophysiological data [33]. This latter observation implies that the steric arrangement and size of the groups attached to the chiral center might be more important in overall activity of the molecule than the carbon double bond (Fig. 2E and 3), since (*R*)-3-octanol comprised half of this racemic blend. In fact, the most effective compounds in our study had a chiral center at the 3-position (Fig. 3).

## Discussion

Here we show for the first time conclusive evidence that an insect OR is capable of enantioselectivity. However, other types of isomer selectivity by ORs have previously been reported. *An. gambiae* ORs exhibit preferences for positional isomers of cresol [38] and a larval OR from *Bombyx mori* discriminates between cis/trans isomers of jasmone [39]. Electrophysiological studies have shown that receptor neurons can respond selectively to enantiomers. For example, the Japanese beetles, *Anomala osakana* and *Popillia japonica*, respond to both enantiomers of the japonilure pheromone with opposite behavioral effects [5]. This behavior is mediated by two different OSNs, localized within the same sensillum, that respond specifically to one enantiomer [40]. It was also shown that pheromone-binding protein (PBP) did not discriminate between the two enantiomers [40]. Similar evidence was advanced in the case of *Cx. quinquefasciatus* in which OSN B in the capitate pegs displayed strong selectivity for (*R*)-(—)-1-octen-3-ol [33].

Lu et al. [16] showed that AgOR8, when expressed in oocytes, was narrowly tuned and responded best to the racemic 1-octen-3-ol among a panel of 82 odorants. Their study indicated the possibility that AgOR8 was capable of enantioselectivity. However, this property was not conclusively demonstrated due to the absence of dose-response curves for the (*R*) and (*S*) forms of 1-octen-3-ol. OSN B in the capitate peg sensillum of *An. gambiae* responds to racemic 1-octen-3-ol and the same neuron in *Cx. quinquefasciatus* discriminates both enantiomers [33]. The presence of the *Or8* gene in both *An. gambiae* and *Ae. aegypti* genomes [37], [41], two species separated by 140-200 million years [42], and the fact that *Cx. quinquefasciatus* is able to discriminate both octenol enantiomers at the physiological level, strongly suggest that the molecular detection mechanism for this kairomone has been conserved in the Culicinae lineage. It remains to test this hypothesis with AgOR8 and the *Cx. quinquesfasciatus* counterpart.

AaOR8 displays sensitivity levels akin to the ones observed between insect pheromone receptors expressed in Xenopus oocytes and their cognate pheromone ligands [24], [43]. The honey bee *Apis mellifera* OR11 (AmOR11) responds to the queen pheromone 9-oxo-2-decenoic acid at the nanomolar range while other AmORs exhibit weak or no response to the same compound [43]. In contrast, *Bombyx mori* OR1 (BmOR1) response to bombykol in the oocyte expression system is in the micromolar range [24].

The most important conclusion from these experiments is that the chiral center is critical for proper recognition of (*R*)-(—)-1-octen-3-ol by AaOR8. AaOR8 exhibits a strong preference towards (*R*)-(—)-1-octen-3-ol and an exquisite degree of selectivity between the two enantiomeric forms, compatible with the notion that the topography of the prospective binding site is complementary to that of (*R*)-(—)-1-octen-3-ol in order to maximize the desired interactions. Chain length and the steric arrangement provided by the chiral center are critical for AaOR8 activation. The differential selectivity of AaOR8 towards both enantiomers of octenol and the loss of sensitivity toward the planar conjugated ketone suggest that one likely interaction involves a hydrogen bond between the oxygen atom of the hydoxyl moiety attached to the chiral center and an amino-acid residue in the receptor binding pocket. Whether the oxygen atom is a hydrogen bond donor or acceptor will have to be determined experimentally.

Our experiments suggest that ORs with “broad” response spectra [44] may actually be narrowly tuned to cognate ligands yet to be discovered and underscore the necessity to test individual enantiomers when chiral odorants are involved. New families of olfactory receptors identified in vertebrates [45] and insects [46] expand the response repertoire for specific ligands whose detection heretofore was assigned to broadly tuned ORs. Moreover, AaOR8 enantioselectivity advocates the shape theory of olfaction over vibrational theories since both octenol enantiomers have identical vibrational signatures but different shapes [47].

While several reports have shown that odorant-binding proteins (OBPs) present in the perireceptor lymph enhance OSN sensitivity [48], [49] and in a few cases participate in odorant specificity [50], most ORs can be activated by odorants directly [51], [52]. DMSO, the organic solvent used in our experiments, has been shown to be as efficient as pheromone-binding proteins (PBPs) at sensitizing OSNs to odorants [49]. As such, DMSO is certainly responsible for the overall activation of these receptors by serving as a carrier thus presenting the tested odorants to them. The dose-response relationships describing the various degrees of sensitivity between AaOR8 and closely related octenol analogues range between 10 and 100-fold. As a constant parameter, the organic solvent DMSO cannot be responsible for the differential sensitivity levels of AaOR8 and notably for its remarkable enantioselective capabilities. Therefore, we propose that part of the sensitivity and most of the specificity toward *(R)*-(—)-1-octen-3-ol is achieved by AaOR8 and these features may not require the assistance of helper proteins such as the OBPs present in the perireceptor lymph. These findings do not necessarily exclude the possibility that OBPs, thought to ferry odorants to the ORs, may be involved in the sensitivity and specificity of the 1-octen-3-ol sensing OSNs.

However, evidence supporting the potential enantioselective properties of OBPs in insects is scant. For example, pheromone receptor neurons of the gypsy moth, *Lymantria dispar*, well discriminate the two enantiomers of the pheromone disparlure [53]. Plettner et al. showed that PBP1 and PBP2 differentiate [54], albeit slightly [55], the two enantiomers of the pheromone. This selectivity was not observed in an earlier study [56]. In fact, a crystallographic study in cockroach indicates that PBP does not discriminate the two enantiomeric pairs of the cockroach pheromone [57]. Our cell expression assay being devoid of OBPs suggests that AaOR8 is sufficient to account for enantioselectivity and is consistent with electrophysiological data gathered from the mosquito *Cx. quinquefasciatus* [33]. These results represent an important step toward understanding enantioselectivity in odorant detection processes. Further, a basis is provided for the utilization of ORs for the discovery of behaviorally and optically active drugs in the same fashion the pharmaceutical field has done for the past 30 years.

## Materials and Methods

### Heterologous Expression of *AaOr7* and *AaOr8* in *Xenopus laevis* Oocytes

*AaOr7* and *AaOr8* cRNAs were synthesized from linearized pSP64DV expression vectors (Dr. Zwiebel, Vanderbilt University) using the mMESSAGE mMACHINE SP6 kit (Ambion). Following mechanical disruption of the Xenopus ovaries, stage V-VII oocytes were treated for 30 min at room temperature under 150 rpm shaking with a 2 mg/mL collagenase (SIGMA, C6895) solution in OR-2 buffer (5 mM HEPES, 1 mM Na_2_HPO_4_, 82.5 mM NaCl, and 2.5 mM MgCl_2_ [pH 7.6]). All procedures were performed in accordance with the NIH Institutional Animal Care and Use Committee and NIH guidelines. Oocytes were subsequently washed 5 times with OR-2 buffer, 5 times with MBSH buffer (10 mM HEPES, 2.4 mM NaHCO_3_, 8.8 mM NaCl, 1 mM KCl, 0.82 mM MgSO_4_, 0.41 mM CaCl_2_ and 0.33 mM (CaNO_3_)_2_, [pH 7.6]), 5 times with MBSH supplemented with 50 µg/ML gentamycin and 5 times with Ringer's buffer (96 mM NaCl, 2 mM KCl, 5 mM MgCl_2_, 6H_2_O, 5 mM HEPES and 0.8 mM CaCl_2_ [pH 7.6]) supplemented with 5% heat-inactivated horse serum, 50 µg/mL tetracycline, 100 µg/mL streptomycin and 550 µg/mL sodium pyruvate. Individual oocytes were allowed to recover overnight prior to injection with 10 ng of each cRNA and were recorded 4 to 6 days post-injection.

### Electrophysiological Recordings

Whole-cell currents were recorded using the two-microelectrode voltage clamp technique [24], [58]. Odorants were dissolved in dimethyl sulfoxide (DMSO) at a 1∶10 ratio so that stock solutions could be made. Prior to recording, stock solutions were diluted in Ringer's solution [pH 7.6] (96 mM NaCl, 2 mM KCl, 5 mM MgCl_2_, 5 mM HEPES and 0.8 mM CaCl_2_) to the indicated concentrations before being applied to Xenopus oocytes in a RC-3Z oocyte recording chamber (Warner Instruments). Oocytes were continuously perfused by either pure Ringer's solution or exposed for 8 sec to serial dilutions of odorants dissolved in Ringer's solution. Odorant-induced currents were recorded with an OC-725C oocyte clamp (Warner Instruments) at a holding potential of −80 mV. Between stimulations, oocytes were allowed to return to their membrane resting potential by washing out the odorants using pure Ringer's solution. Data acquisition and analysis were carried out with Digidata 1440A and pCLAMP10 software (Axon Instruments).

### Data Analysis

Statistical analyses (GraphPad Prism5 Software, Inc.) of the logEC_50_ means were performed using an ordinary one-way ANOVA in conjunction with the Tukey Kramer multiple comparison post test (95% confidence interval). Multiple comparison tests reported by Prism5 do not report exact P values but tell the significance level for each pairwise comparison (see Fig. 2 legend). In all figures, graphical results are shown as means and standard error of the mean for a minimum of six independent oocytes. EC_50_ values for individual compounds were extrapolated using the non-linear regression curve fit function provided in Prism5.

### Materials

3-Octanol (99%) and 1-hepten-3-ol (97%) were obtained from SIGMA. 1-Nonen-3-ol (98%), 1-octen-3-one (97%) and 1-octen-4-ol (99%) were obtained from Alfa Aesar. (*R*)-(—)-1-octen-3-ol (99.6% R) and (*S*)-(+)-1-octen-3-ol (99.9% S) were custom synthesized by Bedoukian Research, Inc.
